# Integrative Sequencing and Proteogenomic Approaches to Intratumoral Heterogeneity in Cholangiocarcinoma: Implications for Precision Diagnosis and Therapy

**DOI:** 10.3390/medsci14010030

**Published:** 2026-01-07

**Authors:** Sirinya Sitthirak, Arporn Wangwiwatsin, Apinya Jusakul, Nisana Namwat, Poramate Klanrit, Sittiruk Roytrakul, Hasaya Dokduang, Thitinat Duangchan, Yanisa Rattanapan, Attapol Titapun, Apiwat Jareanrat, Vasin Thanasukarn, Natcha Khuntikeo, Teh Bin Tean, Luke Boulter, Yoshinori Murakami, Watcharin Loilome

**Affiliations:** 1 Department of Medical Technology, School of Allied Health Sciences, Walailak University, Nakhon Si Thammarat 80160, Thailand; sirinya.sit@wu.ac.th (S.S.); thitinat.du@wu.ac.th (T.D.); yanisa.rt@wu.ac.th (Y.R.); 2 Research Excellence Center for Innovation and Health Products (RECIHP), Walailak University, Nakhon Si Thammarat 80160, Thailand; 3 Department of Systems Biosciences and Computational Medicine, Faculty of Medicine, Khon Kaen University, Khon Kaen 40002, Thailand; arpowa@kku.ac.th (A.W.); nisana@kku.ac.th (N.N.); porakl@kku.ac.th (P.K.); 4 Cholangiocarcinoma Research Institute, Khon Kaen University, Khon Kaen 40002, Thailand; apinjus@kku.ac.th (A.J.); hasaya.d@msu.ac.th (H.D.); attati@kku.ac.th (A.T.); apiwat.apj@kku.ac.th (A.J.); maxpasin34@gmail.com (V.T.); natckh@kku.ac.th (N.K.); 5 Faculty of Associated Medical Sciences, Khon Kaen University, Khon Kaen 40002, Thailand; 6 National Center for Genetic Engineering and Biotechnology (BIOTEC), National Science and Technology Development Agency (NSTDA), Pathum Thani 12120, Thailand; sittiruk@biotec.or.th; 7 Faculty of Medicine, Mahasarakham University, Mahasarakham 44000, Thailand; 8 Hematology and Transfusion Science Research Center, Walailak University, Nakhon Si Thammarat 80160, Thailand; 9 Department of Surgery, Faculty of Medicine, Khon Kaen University, Khon Kaen 40002, Thailand; 10 National Cancer Centre Singapore, Duke-NUS Medical School, Singapore 169857, Singapore; gmstbt@nus.edu.sg; 11 MRC Human Genetics Unit, Institute of Genetics and Cancer, The University of Edinburgh, Western General Hospital, Crewe Road South, Edinburgh EH4 2XU, Scotland, UK; luke.boulter@ed.ac.uk; 12 Department of Molecular Biology, Institute for Advanced Medical Sciences, Nippon Medical School, Tokyo 113-8602, Japan; yoshinori-murakami@nms.ac.jp

**Keywords:** cholangiocarcinoma, clonal evolution, intratumoral heterogeneity, mass spectrometry, multi-omics, proteogenomics, tumor microenvironment

## Abstract

Cholangiocarcinoma (CCA) is a highly aggressive cancer of the biliary tract, distinguished by significant intratumoral heterogeneity (ITH), which contributes to therapy resistance and unfavorable clinical outcomes. Traditional genome profiling has revealed recurring driver changes in CCA; yet, genomic data alone fails to elucidate functional pathway activation, adaptive signaling, and the diverse treatment responses reported among tumor locations and disease subtypes. This review analyses the use of integrated sequencing technologies, proteogenomics, and phosphoproteomics to systematically characterize intratumoral heterogeneity in cholangiocarcinoma and convert molecular diversity into therapeutically applicable discoveries. We present evidence that the combination of genomic sequencing and mass spectrometry–based proteomics facilitates the direct correlation of genetic mutations with protein expression, post-translational modifications, and signaling system activity. Phosphoproteomic profiling specifically offers functional insights into kinase-driven networks that dictate tumor aggressiveness, therapeutic susceptibility, and adaptive resistance mechanisms, which cannot be anticipated only from DNA-level analysis. We propose that integrating proteogenomic and phosphoproteomic analyses into diagnostic and therapeutic assessments can enhance molecular classification, reveal subtype- and region-specific therapeutic dependencies, and guide rational combination treatment strategies, based on recent extensive proteogenomic studies and functional proteomic investigations in CCA. Pathway-level analysis of intratumoral heterogeneity provides a framework for selecting targeted medicines, predicting resistance, and informing personalized treatment strategies in CCA. The combination of sequencing, proteogenomics, and phosphoproteomics is essential for advancing precision oncology in cholangiocarcinoma. The implementation of this multi-layered analytical approach may better patient classification, refine therapy choices, and eventually improve clinical outcomes for individuals with this particular heterogeneous cancer.

## 1. Introduction

Cholangiocarcinoma (CCA) is an aggressive malignancy arising from the epithelial cells of the bile ducts, constituting roughly 10–15% of all primary liver malignancies globally [1,2]. The disease is famously difficult to diagnose in its early stages, resulting in most patients presenting with advanced-stage cancer, which leads to a poor prognosis and few treatment alternatives [2]. Although progress has been made in surgical methods and chemotherapy, the overall survival rate for CCA remains low, mostly because of significant ITH, which hinders effective treatment approaches [3].

ITH indicates the existence of genetically, epigenetically, and phenotypically different subpopulations of cancer cells within an individual tumor [4,5]. This variability is influenced by a combination of genetic mutations, including abnormalities in *TP53*, *KRAS*, and *IDH1*, and epigenetic modifications such as DNA methylation and histone alterations [6,7]. Furthermore, interactions within the tumor microenvironment (TME), comprising stromal cells, immune cells, and extracellular matrix components, substantially influence the selection of certain cancer cell clones, hence increasing heterogeneity [8,9].

The idea of clonal evolution, characterized by the emergence of subclonal populations with unique mutational patterns, has proven pivotal in comprehending ITH in cancer. In this paradigm, early changes common to all cancer cells are referred to as trunk mutations, but subsequent, more particular modifications are designated as branch mutations. The clonal variety inside a tumor influences its progression and treatment response, rendering it a vital factor in the formulation of effective therapeutic methods [10,11].

The development of powerful multi-omics methodologies, especially proteogenomics, has equipped researchers with robust instruments to elucidate the intricate molecular framework of CCA. Proteogenomics amalgamates proteome data from MS with genomic information derived from next-generation sequencing (NGS), facilitating an exhaustive examination of the molecular characteristics that underpin tumor heterogeneity. MS-based proteomics has facilitated the detection of differently expressed proteins, post-translational changes, and proteoforms, which are essential for comprehending the functional implications of genetic variations [12,13,14,15].

Despite the rising volume of data, several features of ITH in CCA remain poorly understood, notably about how this heterogeneity promotes disease progression and therapy resistance. This review seeks to conduct a comprehensive analysis of the mechanisms underlying ITH in CCA, the function of MS in proteogenomics research, and the ramifications of these discoveries for personalized therapy. By investigating recent advancements in multi-omics technologies and their application to CCA, we aim to illuminate the possibility for innovative therapeutic options that can more effectively tackle the obstacles presented by tumor heterogeneity [16,17].

Recent advancements in sequencing technologies, proteogenomics, and phosphoproteomics enable a transition from mere descriptive tumor profiling to a functionally informed framework for the diagnostic and therapeutic assessment of CCA, especially considering the significant ITH. Genomic sequencing identifies driver mutations and clonal architecture, whereas proteogenomic integration facilitates the direct evaluation of how these genetic modifications influence protein expression, post-translational modifications, and pathway activity, which ultimately govern tumor behavior and treatment response [18,19].

Extensive proteogenomic investigations have shown that CCA can be categorized into biologically and clinically significant subtypes through the integration of genomic and proteomic characteristics, uncovering subtype-specific signaling dependencies and actionable therapeutic vulnerabilities that are not discernible from DNA data alone [18,19,20]. These investigations emphasize that ITH is evident not only in mutational variations but also in diverse protein expression patterns and dynamic signaling networks within tumor areas, highlighting the need for pathway-level analysis when assessing treatment targets.

Phosphoproteomics enhances this approach by documenting the real-time activation status of oncogenic signaling pathways that promote tumor aggressiveness, adaptability, and medication resistance. Integrated proteomic and phosphoproteomic analyses indicate that unique kinase activities and compensatory signalling mechanisms can characterize aggressive disease phenotypes, as evidenced in biliary tract malignancies, including gallbladder cancer, which exhibits molecular and signaling characteristics similar to CCA [21,22]. These findings substantiate the notion that phosphoproteomic analyses are crucial for identifying functionally active pathways and formulating sensible combination therapy in diverse tumors.

In addition to extensive cohort-based investigations, novel functional and spectroscopic methodologies underscore the translational significance of including molecular layers. Recent research integrating FTIR microspectroscopy with in vitro and in silico analyses demonstrates the ability to capture molecular phenotypes related to drug response at a functional level in CCA models, underscoring the significance of multi-modal approaches for comprehending tumor heterogeneity and therapeutic mechanisms [23].

Collectively, these investigations establish a robust basis for reconfiguring the integration of sequencing technologies, proteogenomics, and phosphoproteomics into a cohesive analytical framework for CCA. Instead of functioning as separate technologies, their integrated use facilitates thorough characterization of ITH ranging from clonal genetic modifications to functional signaling states thus enhancing diagnostic classification, biomarker identification, and personalized treatment approaches adapted to the diverse characteristics of CCA [18,19,21,23].

## 2. Mechanisms Contributing to Tumor Heterogeneity

**Figure 1:**
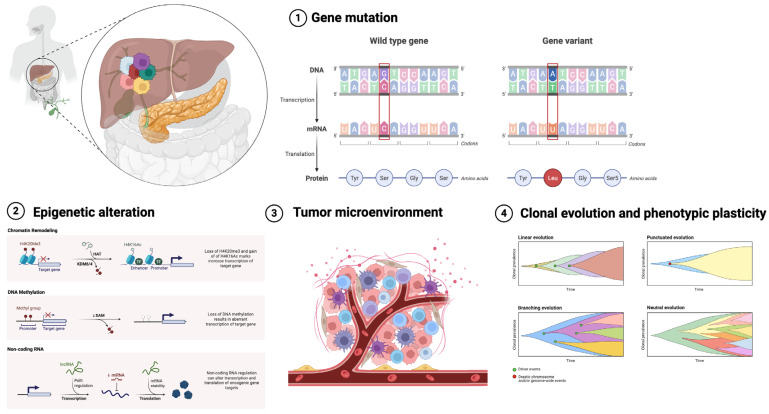
Mechanisms that contribute to tumor heterogeneity in cancer. Tumor heterogeneity emerges from various interrelated causes, such as genetic mutations, epigenetic modifications, phenotypic plasticity, and the tumor microenvironment. Genetic mutations propel clonal evolution, with significant mutations common across tumor locations and others unique to specific regions. Epigenetic modifications, including histone alterations, govern gene expression without modifying the DNA sequence. Phenotypic plasticity allows tumor cells to shift among epithelial, intermediate, and mesenchymal states, influenced by elements within the tumor microenvironment, including immune cells, cancer-associated fibroblasts (CAFs), extracellular matrix (ECM), and cytokines. Collectively, these pathways generate intricate ITH that influences tumor growth and resistance to therapy.

ITH is a defining characteristic of CCA and constitutes a significant obstacle to effective treatment. ITH’s complexity stems from various variables shown in Figure 1, such as genetic mutations, epigenetic changes, and the impact of the tumor microenvironment. These factors interact dynamically, propelling the clonal development of tumor cells and contributing to varied phenotypic characteristics within a single tumor [5].

### 2.1. Genetic Mutations

Genetic mutations are a primary catalyst of ITH in CCA. Tumor cells obtain somatic mutations that facilitate clonal proliferation, producing genetically different subpopulations. Mutations can be categorized into trunk mutations, which manifest early in tumorigenesis and are present in all cancer cells, and branch mutations, which develop thereafter and are confined to particular subclonal populations [24,25]. Frequent genetic modifications in CCA encompass mutations in critical oncogenes and tumor suppressor genes, including *TP53*, *KRAS*, and *IDH1*. These alterations are crucial in tumor genesis, development, and therapeutic response, with branch mutations enhancing clonal diversity and therapeutic resistance [6,24,26].

Recent studies have emphasized the significance of clonal evolution in influencing tumor heterogeneity. Clonal evolution entails the selection of subclones possessing beneficial mutations that enhance their fitness, enabling them to surpass competing cell populations within the tumor. This continuing selection pressure leads to the formation of new subclonal populations with unique mutational patterns, further boosting the genetic complexity of the tumor [8,27].

### 2.2. Epigenetic Alterations

In addition to genetic mutations, epigenetic alterations significantly contribute to ITH in CCA. Epigenetic modifications, such as DNA methylation, histone alterations, and chromatin restructuring, affect gene expression without modifying the fundamental DNA sequence. These modifications can be transmitted throughout cellular divisions, resulting in consistent phenotypic variability among tumor cells [5,28].

Atypical DNA methylation patterns are frequently identified in CCA and correlate with the repression of tumor suppressor genes. Hypermethylation of promoter regions in genes like CDKN2A has been associated with increased cancer cell proliferation and survival [29,30]. Histone changes, including as acetylation and methylation, are essential in modulating chromatin architecture and gene expression. Dysregulation of histone-modifying enzymes, such as EZH2, can result in modified expression of oncogenes and tumor suppressors, hence leading to epigenetic heterogeneity within the tumor [31].

The interaction between genetic mutations and epigenetic modifications can establish a feedback loop that enhances tumor heterogeneity. Mutations in genes that encode chromatin modifiers, like as ARID1A, are commonly found in CCA and can result in extensive epigenetic dysregulation. These modifications augment the flexibility of cancer cells, enabling them to acclimate to environmental stresses and treatment strategies [32,33].

### 2.3. Tumor Microenvironment

The tumor microenvironment (TME) is a dynamic and intricate ecology that significantly influences intratumoral heterogeneity. It comprises several cellular types, including cancer-associated fibroblasts (CAFs), immunological cells, endothelial cells, and components of the extracellular matrix. The interactions between tumor cells and the tumor microenvironment facilitate phenotypic plasticity and encourage the selection of aggressive subclones [34].

CAFs are essential constituents of the TME and significantly influence tumor dynamics. CAFs can affect the composition of the extracellular matrix, release growth factors, and facilitate angiogenesis, all of which contribute to the development of various tumor cell populations [35]. Moreover, immune cells, especially tumor-infiltrating lymphocytes (TILs), display considerable heterogeneity inside the TME. The variability in the composition and functional condition of tumor-infiltrating lymphocytes (TILs) has been linked to variations in immune response and treatment outcomes in individuals with CCA [36,37,38].

Hypoxia, a prevalent characteristic of the tumor microenvironment, can exacerbate ITH by promoting differential gene expression and favoring cancer cell subpopulations with improved survival traits. Hypoxic circumstances stimulate the activation of hypoxia-inducible factors (HIFs), which govern the expression of genes associated with angiogenesis, metabolism, and cellular survival. The selection pressure induced by hypoxia can result in the proliferation of more aggressive cancer cell clones, hence facilitating tumor development and resistance to therapy [39].

### 2.4. Clonal Evolution and Phenotypic Plasticity

Clonal evolution, along with the effects of the tumor microenvironment and epigenetic modifications, leads to a markedly diverse tumor landscape. Phenotypic plasticity, the capacity of cancer cells to transition between several states, exacerbates the comprehension of ITH. For instance, epithelial-to-mesenchymal transition (EMT) is a process that contributes to cellular plasticity, allowing epithelial cancer cells to acquire mesenchymal features, which are associated with greater invasiveness and resistance to therapies [17,40].

The interaction of genetic, epigenetic, and microenvironmental variables emphasizes the intricacy of ITH in CCA and reinforces the necessity for comprehensive multi-omics strategies to precisely describe tumor heterogeneity. Proteogenomics, which amalgamates proteomic and genomic data, has arisen as a valuable instrument for elucidating the molecular foundations of intratumoral heterogeneity, providing novel insights into prospective therapeutic targets and techniques for individualized cancer treatment [16,41,42].

## 3. Mass Spectrometry in Proteogenomics

MS has emerged as a fundamental tool in proteomics, facilitating extensive investigation of protein expression, changes, and interactions on an unparalleled scale. In proteogenomics, MS is essential for combining proteomic data with genomic information, enhancing the comprehension of the molecular landscape of malignancies like CCA. This methodology enables researchers to discern proteoforms, post-translational changes, and new protein variations associated with genetic mutations, providing significant insights into the functional implications of tumor heterogeneity [18,43,44].

### 3.1. High Sensitivity and Throughput

A primary benefit of contemporary MS is its exceptional sensitivity, enabling the identification of low-abundance proteins that may be pivotal in tumor biology. Advanced MS methods, like Orbitrap and time-of-flight (TOF) analyzers, provide excellent resolution and precision, facilitating the identification of hundreds of proteins from minimal sample volumes. The amalgamation of liquid chromatography (LC) with MS significantly improves the separation of intricate protein mixtures, enabling the identification of a diverse array of peptides, including those exhibiting post-translational changes [45,46].

Recent advancements in MS technology, such as enhanced scan speeds and optimized ion transmission, have markedly increased the throughput of proteome investigations. Cutting-edge mass spectrometry devices can now execute swift data acquisition with high mass precision, enabling the concurrent identification and quantification of thousands of proteins across various samples. This high throughput capability is particularly advantageous for proteogenomics research, which generally involve the analysis of large cohorts to capture the various genetic profiles of different tumor subpopulations [47,48].

### 3.2. Unbiased Detection and Quantification

A significant advantage of mass spectrometry in proteogenomics is its ability to detect and quantify proteins in a generally unbiased manner, without requiring prior knowledge of specific molecular targets. In contrast to antibody-based techniques, which are constrained by the availability and specificity of reagents, mass spectrometry (MS) utilizes the mass-to-charge (*m*/*z*) ratio of ions, allowing for the direct measurement of peptides and proteins based on their intrinsic physical properties [49].

Mass spectrometers segregate ions based on their mass-to-charge (*m*/*z*) ratios, facilitating the identification of both established and novel proteins, even those with uncommon or unforeseen mutations. This unbiased identification is particularly helpful in the context of researching intratumoral heterogeneity, where novel protein variations may develop due to genetic mutations or alternative splicing events. Additionally, the great dynamic range of contemporary MS instruments allows for the simultaneous measurement of both high-abundance and low-abundance proteins, providing a full proteomic profile of the tumor [43].

### 3.3. Applications in Phosphoproteomics

Phosphoproteomics, a subdivision of proteomics dedicated to the examination of phosphorylated proteins, has emerged as a crucial instrument in proteogenomics research. Phosphorylation is a crucial post-translational alteration that governs various physiological functions, such as signal transmission, cell cycle progression, and apoptosis. Atypical phosphorylation patterns are commonly detected in cancer and are associated with the deregulation of essential signaling pathways, including PI3K/AKT and MAPK [50,51,52].

Mass spectrometry-based phosphoproteomics facilitates the identification and measurement of phosphorylation sites throughout the proteome, offering insights into the functional status of signaling networks inside the tumor. Phosphoproteomic research in CCA has shown modifications in critical signaling pathways that facilitate tumor development and treatment resistance. The capacity to precisely map phosphorylation sites has enabled the identification of new biomarkers and prospective therapeutic targets, hence advancing the creation of tailored medicines [53,54].

### 3.4. Integration with Genomic Data

The amalgamation of MS-based proteomics with genetic data constitutes the essence of proteogenomics. By integrating proteomic profiles with genomic data from NGS, researchers can directly associate changes in protein expression with underlying genetic modifications. This integrated technique facilitates the detection of novel protein variants, including those arising from somatic mutations, splice variants, and gene fusions, which may remain undetectable by genomic analysis alone [55,56].

**Figure 2:**
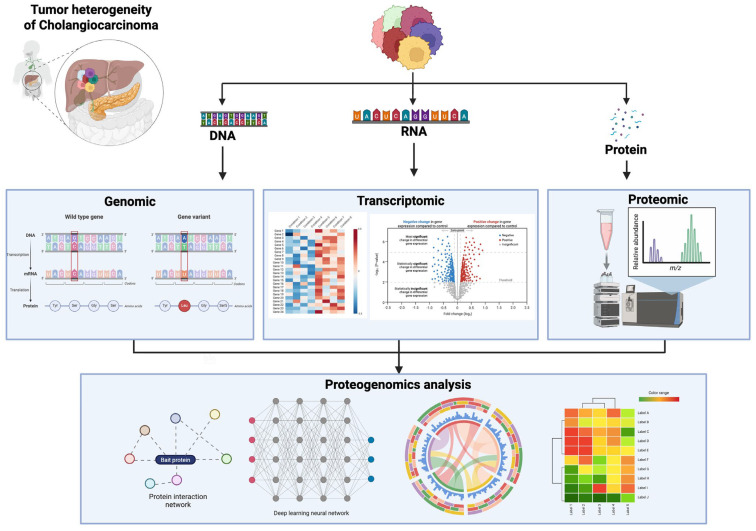
Multi-omics integration in proteogenomics. Genomics reveals mutations affecting protein sequences. Transcriptomics highlights differential gene expression. Proteomics and phosphoproteomics identify and quantify proteins and post-translational modifications. Combined, proteogenomics integrates these layers to map molecular interactions and networks for comprehensive biological insights.

Proteogenomics gives a comprehensive perspective of the tumor’s molecular landscape (Figure 2), illustrating how genomic mutations materialize at the protein level and how these changes lead to ITH. In CCA, proteogenomic investigations have shown unique protein expression profiles linked to particular genetic abnormalities, offering insights into the mechanisms behind tumor growth and therapeutic resistance [7,57,58].

### 3.5. Technological Advancements and Future Directions

The rapid advancement of MS technologies perpetually enhances the potential of proteogenomics. Innovations such as ion mobility spectrometry, enhanced fragmentation methods, and improved data acquisition algorithms have further increased the depth and accuracy of proteomic analyses. Ion mobility spectrometry, for instance, adds an additional dimension of separation based on the size and shape of ions, enhancing the resolution of complicated peptide combinations [46,59].

Future directions in proteogenomics include combining multi-omics data, encompassing transcriptomics and metabolomics, to construct a more thorough comprehension of the molecular foundation of tumor heterogeneity. Advances in computer tools and machine learning algorithms are also expected to assist the processing and interpretation of proteogenomic data, aiding the discovery of novel biomarkers and treatment targets [60,61].

### 3.6. Practical Strengths and Limitations of Mass Spectrometry-Based Proteogenomics in Cholangiocarcinoma

MS-based proteogenomics provides a functional connection between genomic alterations and their phenotypic outcomes in CCA, facilitating pathway- and protein-level analysis of ITH that cannot be accurately deduced from DNA alone [13,14]. A key advantage is the direct quantification of protein abundance, proteoforms, and post-translational modifications throughout tumor regions, facilitating the identification of subtype- or region-specific signaling dependencies and therapeutic vulnerabilities pertinent to precision oncology in CCA [18,19]. Moreover, MS-based readouts can elucidate the translation of canonical genomic events into actionable biology, offering a clinically relevant perspective on heterogeneity when genetic drivers are conserved but functional pathway activation varies across spatial tumor compartments [14,15].

Nonetheless, proteogenomics possesses distinct limitations that are especially pertinent in CCA. Clinical translation is limited by the necessity for adequate tissue quantity and quality; this is particularly problematic in unresectable cases where biopsy material is scarce or stromal content is elevated, which may obscure tumor-derived signals and complicate the interpretation of tumor-microenvironment interactions [15,62]. Secondly, technical variability in sample preparation, mass spectrometry acquisition, and bioinformatic pipelines can influence reproducibility, underscoring the necessity for standardized workflows and stringent quality control for proteogenomic outputs to be utilized in patient stratification [15,63]. Third, although proteogenomics is proficient in elucidating functional output, it is inferior to sequencing for the thorough identification of low-frequency subclonal variants, structural rearrangements, or copy-number alterations that characterize clonal architecture, which are crucial for interpreting ITH in CCA [57,64].

Recent extensive research within the framework of CCA illustrates the distinct advantages of proteogenomic integration for enhancing molecular categorization and uncovering treatment possibilities that surpass genomics-only methodologies [18,19]. Thus, MS-based proteogenomics serves optimally as an auxiliary component that functionally annotates sequencing-defined heterogeneity, rather than supplanting genomic profiling in CCA care pathways [14,15].

## 4. Sequencing Technologies in Cancer Research

The development of high-throughput sequencing tools has transformed cancer research, facilitating comprehensive analysis of the genetic landscape of malignancies. In the area of CCA, NGS has been essential in revealing the intricate molecular heterogeneity inherent to this cancer. By providing full genomic data, these methods have allowed researchers to discover critical driver mutations, detect novel gene fusions, and explore the clonal architecture of cancers [64,65].

### 4.1. Evolution of Sequencing Technologies

**Figure 3:**
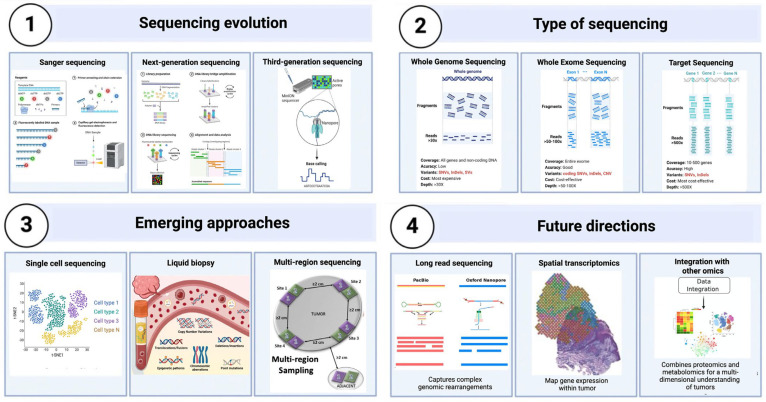
Overview of sequencing technologies and emerging approaches in genomic analysis. (1) Evolution of Sequencing: A chronological overview of sequencing technologies, beginning with Sanger sequencing, which employs chain termination chemistry, progressing to next-generation sequencing (NGS) characterized by massively parallel library amplification, and culminating in third-generation sequencing, including nanopore-based technologies that facilitate real-time base calling. (2) Types of Sequencing: A comparative examination of primary sequencing methodologies, encompassing whole genome sequencing (WGS) for extensive genomic coverage, whole exome sequencing (WES) aimed at coding areas, and targeted sequencing concentrating on specific genes or regions for in-depth analysis. (3) Emerging Methodologies: Advanced methodologies in genomic research, including single-cell sequencing for elucidating cellular heterogeneity, liquid biopsy for non-invasive biomarker identification, and multi-region tumor sample for spatial heterogeneity assessment. (4) Future Directions: Progress in long-read sequencing for structural variation identification, spatial transcriptomics for gene expression localization within tissues, and multi-omics integration merging proteomics and metabolomics for comprehensive tumor profiling.

Sequencing technologies have grown significantly in recent decades (Figure 3), transitioning from conventional Sanger sequencing to more advanced methods, including whole-genome sequencing (WGS), whole-exome sequencing (WES), and targeted sequencing panels. Sanger sequencing, while exceptionally precise, is constrained by its poor throughput and elevated cost, rendering it unsuitable for extensive cancer research. In contrast, NGS allows massively parallel sequencing, allowing for the simultaneous examination of millions of DNA fragments. This capability has markedly enhanced the speed and reduced the cost of sequencing, establishing it as the preferred method for cancer genomics research [66,67].

Whole-genome sequencing (WGS) offers a comprehensive analysis of the entire genome, encompassing all forms of genetic variations, such as single nucleotide variants (SNVs), insertions, deletions, copy number variations, and structural rearrangements. Although whole genome sequencing (WGS) provides the most broad coverage, its prohibitive cost and substantial data needs sometimes restrict its application in clinical environments. Whole-exome sequencing (WES), which targets the protein-coding sections of the genome (the exome), offers a more economical option while effectively identifying the bulk of known disease-related mutations [68,69].

Targeted sequencing panels, which concentrate on particular cancer-associated genes, provide an efficient method for identifying clinically significant alterations. These panels are particularly valuable in translational research and clinical diagnostics, where rapid, focused examination is needed to guide therapeutic decisions. However, focused panels may overlook fresh or unexpected mutations beyond the designated gene set, limiting their ability to properly define tumor heterogeneity (Figure 3) [70].

### 4.2. Limitations of Sequencing Technologies

Despite their benefits, contemporary sequencing technologies encounter numerous obstacles that may affect the precise characterization of tumor heterogeneity. A significant restriction is the problem of sampling bias, particularly when employing single-region biopsies. Tumors have inherent heterogeneity, and a solitary biopsy may fail to encapsulate the whole genetic diversity inside the tumor, resulting in an incomplete or inaccurate representation of its clonal composition [62,71].

A further challenge resides in the interpretation of sequencing data, especially in differentiating between driver mutations, which facilitate tumorigenesis, and passenger mutations, which are incidental and do not influence tumor behavior. This differentiation is essential for recognizing actionable mutations suitable for therapeutic targeting. Moreover, the existence of subclonal mutations alterations seen exclusively in a fraction of cancer cells complicates the investigation as these mutations may be crucial for comprehending treatment resistance and tumor growth [72,73].

### 4.3. Emerging Sequencing Approaches

Recent advancements in sequencing technologies have developed novel methodologies to mitigate some constraints. Single-cell sequencing has emerged as a potent method for analyzing tumor heterogeneity at the cellular level. Single-cell sequencing offers exceptional resolution by studying the genome, transcriptome, or epigenome of individual cells, uncovering the diversity of cell populations within a tumor and discovering unusual subclones that may contribute to disease progression or therapeutic resistance [28,74,75].

Furthermore, methodologies like multi-region sequencing and liquid biopsy have become prominent in cancer research. Multi-region sequencing entails sampling various tumor regions, offering a more thorough understanding of its clonal architecture and mitigating the risk of sampling bias. Liquid biopsy, which examines circulating tumor DNA (ctDNA) in blood samples, provides a non-invasive method for monitoring tumor progression and identifying minimal residual illness. This method is especially beneficial for monitoring alterations in tumor heterogeneity over time and evaluating therapeutic response (Figure 3) [76,77].

### 4.4. Criteria for Selecting Sequencing Technologies

The choice of sequencing technique depends on various aspects, including the research topic, the clinical environment, and the available resources. Whole-genome sequencing is appropriate for discovery-driven research where the goal is to detect novel genetic changes. Whole-exome sequencing is generally preferred in clinical settings due to its mix of extensive coverage and cost-effectiveness. Targeted sequencing panels are appropriate for instances requiring the rapid identification of certain known mutations to guide treatment decisions [78].

Single-cell sequencing is the preferred technique for investigating ITH and clonal evolution, whereas liquid biopsy is advantageous for real-time assessment of tumor dynamics. Ultimately, the integration of several sequencing methodologies, including the combination of bulk sequencing with single-cell or multi-region sequencing, can yield a more comprehensive understanding of the genomic landscape of CCA and improve our insight into the mechanisms behind tumor heterogeneity [79,80].

### 4.5. Future Directions in Sequencing

The future of cancer research depends on the ongoing advancement and utilization of state-of-the-art sequencing technologies. Emerging technologies, including long-read sequencing and spatial transcriptomics, show considerable potential for capturing complicated genomic rearrangements and tracking the spatial structure of gene expression within tumors. Furthermore, improvements in computational techniques and machine learning are anticipated to augment the processing and interpretation of extensive sequencing data, thereby aiding in the discovery of new biomarkers and therapeutic targets [81,82].

The advancement of sequencing technologies, coupled with their integration with other omics data such as proteomics and metabolomics, is essential for developing a thorough multi-dimensional comprehension of tumor biology. This integrative approach, crucial to proteogenomics, could revolutionize the diagnosis and treatment of CCA, facilitating more effective, customized therapy methods (Figure 3) [83,84].

### 4.6. Definitive Pros and Cons of Sequencing Approaches for Analyzing Intratumoral Heterogeneity in Cholangiocarcinoma

Sequencing methods establish the essential framework for elucidating the genetic factors contributing to CCA heterogeneity, encompassing driver changes, clonal architecture, and mutational mechanisms. WGS provides an extensive perspective on genomic variation, encompassing structural variants and non-coding modifications that may influence oncogenesis and evolution; nonetheless, prohibitive costs, computational demands, and extended clinical turnaround times hinder its routine implementation, particularly in contexts necessitating swift targeted actionable results [69,70]. WES is frequently more pragmatic and has been extensively utilized for identifying coding variants associated with therapeutic targets and prognostic stratification; however, it may overlook critical rearrangements and intricate structural alterations pertinent to certain biliary tract oncogenic occurrences [68,69].

Targeted sequencing panels offer benefits for clinical application, including rapidity, cost-effectiveness, and sensitivity to specific actionable mutations; however, they inherently restrict discovery, may inadequately represent rare or unforeseen drivers, and can fail to encompass the full spectrum of intratumoral heterogeneity, especially when subclonal diversity exceeds the targeted gene set [64,70]. Sampling bias is a fundamental constraint in CCA across all bulk sequencing methodologies. Single-region biopsies may fail to capture the spatial heterogeneity of primary tumors or the dynamics of metastatic evolution, resulting in the under-detection of branch mutations and inaccurate calculation of actionable subclones [62,71].

Novel methodologies mitigate these constraints yet present new compromises. Multi-region sequencing enhances the representation of spatial heterogeneity and clonal architecture; however, it necessitates sufficient surgical specimens and systematic sampling methodologies, which may pose challenges in specific anatomical subtypes or advanced disease stages [11,62]. Liquid biopsy provides a less invasive method to assess temporal heterogeneity and treatment response; however, inconsistent ctDNA shedding, assay sensitivity, and the inability to pinpoint signals to specific tumor locations may restrict interpretability [76,80]. Single-cell sequencing can elucidate cellular heterogeneity and identify rare subclones; nevertheless, it is resource-intensive and susceptible to technical noise, dropout, and intricate analytical demands, which currently limit its widespread clinical application in CCA [28,74].

Sequencing is essential for identifying CCA genetic drivers and clonal development; however, it cannot directly assess pathway function or adaptive signaling that influences treatment sensitivity. This disparity necessitates the amalgamation of proteogenomics and phosphoproteomics to convert genetic variability into functional, implementable therapeutic approaches [14,18,19,64].

## 5. Phosphoproteomics and Its Applications

Phosphoproteomics, a distinct subset of proteomics, concentrates on the thorough examination of phosphorylated proteins. Phosphorylation is a pivotal post-translational modification (PTM) in cellular signaling, governing numerous biological processes such as cell proliferation, differentiation, death, and responses to external stimuli. In cancer, abnormal phosphorylation signifies dysregulated signaling pathways, rendering phosphoproteomics an effective instrument for comprehending tumor biology and pinpointing possible treatment targets [85,86,87].

### 5.1. Principles of Phosphoproteomics

Phosphoproteomics encompasses the extraction and detection of phosphorylated peptides by MS. Due to the low abundance of phosphorylated peptides in complex biological materials, enrichment techniques including immobilized metal affinity chromatography (IMAC) and metal oxide affinity chromatography (MOAC) are utilized to preferentially isolate phosphorylated entities [88]. Following enrichment, the phosphorylated peptides are analyzed by tandem MS (MS/MS), which provides high-resolution spectra for the identification and localization of phosphorylation sites. This high sensitivity and specificity allow researchers to map phosphorylation events across the proteome, revealing dynamic changes in signaling networks [88,89,90].

### 5.2. Insights from Phosphoproteomics in Cancer Research

Phosphoproteomics has become a crucial technique in cancer research, bringing unique insights into the dysregulated signaling pathways that cause tumor growth. In CCA, research has employed phosphoproteomics to investigate modifications in critical signaling pathways, including the PI3K/AKT/mTOR and MAPK pathways. These pathways are often hyperactivated in CCA because of mutations or overexpression of upstream receptor tyrosine kinases (RTKs), resulting in enhanced cell proliferation, survival, and migration [53,91,92].

Recent phosphoproteomic investigations have revealed unique phosphorylation patterns linked to particular genetic changes in CCA. Tumors with mutations in the IDH1 gene demonstrate modified phosphorylation of critical enzymes in metabolic pathways, indicating the influence of these mutations on cellular metabolism and energy regulation. These findings offer significant insights into the molecular mechanisms of CCA and underscore possible targets for therapeutic intervention [53,91,92,93].

### 5.3. Applications of Phosphoproteomics in Drug Discovery

Phosphoproteomics is essential for the identification and confirmation of pharmacological targets, especially in targeted cancer therapy. By delineating the phosphorylation landscape of cancer cells, researchers can pinpoint aberrantly active kinases that may function as viable therapeutic targets. In CCA, many kinases, such as *FGFR2*, *EGFR*, and *MET*, have been recognized as major mediators of oncogenic signaling. Phosphoproteomic profiling has enabled the identification of these kinases as prospective therapeutic targets, resulting in the creation of small chemical inhibitors and monoclonal antibodies designed to limit their activity [94,95,96].

Moreover, phosphoproteomics is crucial for elucidating the mechanisms underlying drug resistance. In numerous malignancies, including CCA, resistance to targeted therapy frequently occurs due to compensatory activation of alternative signaling pathways. Inhibition of the PI3K/AKT pathway may result in the overexpression of the MAPK pathway, enabling cancer cells to circumvent the treatment’s inhibitory effects. Phosphoproteomic analysis can elucidate adaptive signaling alterations, facilitating the development of combination medicines that concurrently target numerous pathways to surmount resistance [32,97,98].

### 5.4. Integrative Approaches in Phosphoproteomics

The integration of phosphoproteomics with other omics data, including genomes and transcriptomics, improves our comprehension of the intricate regulatory networks in cancer. Proteogenomics integrates proteomic data with genomic sequencing, facilitating the identification of phosphorylation events with particular genetic changes. This integrative method has proven especially effective in CCA, where it has discerned phosphorylation signals linked to specific genetic subgroups, hence enhancing the understanding of tumor heterogeneity [24,53,58].

A recent study integrated phosphoproteomic and genomic data to examine the function of the EGFR pathway in CCA. The research demonstrated that tumors with amplified EGFR displayed heightened phosphorylation of downstream signaling proteins, including STAT3 and ERK1/2, associated with greater tumor cell proliferation and survival. These findings clarify the functional implications of EGFR amplification and propose possible biomarkers for identifying patients who could benefit from EGFR-targeted treatments [94,99].

### 5.5. Future Directions in Phosphoproteomics

The field of phosphoproteomics is rapidly progressing, propelled by advancements in mass spectrometry equipment and data analysis methods. Novel methodologies, such ion mobility spectrometry and data-independent acquisition (DIA), are enhancing the depth and precision of phosphoproteomic investigations, facilitating the identification of low-abundance phosphorylation events that were once challenging to detect [100].

Future investigations in phosphoproteomics are anticipated to concentrate on enhancing sensitive enrichment techniques and creating innovative computational tools for the integration of multi-omics data. In CCA, these developments will enhance comprehension of the signaling networks that underpin tumor heterogeneity and establish a basis for identifying novel treatment targets. Utilizing phosphoproteomics enables researchers to reveal essential insights into the dynamic control of cancer signaling pathways, hence facilitating the creation of more effective, customized treatment regimen [101,102,103].

### 5.6. Strengths and Limitations of Phosphoproteomics for Functional Stratification in CCA

Phosphoproteomics offers a direct assessment of signaling activity, facilitating the functional classification of tumors according to the activation states of kinase pathways, which frequently contribute to aggressiveness, drug sensitivity, and adaptive resistance. In CCA, this is especially pertinent as tumors with common genomic drivers may demonstrate varied pathway activation across regions, and compensatory signaling can arise during treatment, both of which are optimally assessed at the phosphorylation level [53,87]. Moreover, phosphoproteomics can discern active kinases and signaling modules that may be actionable, even in the absence of discernible targetable genomic alterations, hence broadening the scope of precision medicine options [53,103].

Nonetheless, phosphoproteomics presents limitations that must be expressly acknowledged for CCA applications. Phosphopeptides are typically low in quantity and necessitate enrichment, so augmenting technical difficulty and potentially introducing bias; the depth of data and repeatability are significantly influenced by sample handling and platform standardization [87,88]. Secondly, phosphorylation is dynamic and context-dependent, indicating that pre-analytical variables, ischemia duration, and tissue processing can significantly influence the assessed signaling states, creating challenges for inter-cohort comparisons and clinical application [87,103]. Third, phosphoproteomic analyses generally necessitate considerable tissue input, potentially restricting their applicability in small biopsies prevalent in advanced CCA, and interpretation may be complicated by stromal and immune factors in desmoplastic tumors unless accompanied by meticulous design and supplementary omics layers [15,62].

From a translational perspective, phosphoproteomics is most effective when combined with sequencing and proteogenomics. Sequencing delineates the genomic foundation of ITH, proteogenomics correlates genomic variation with protein expression, and phosphoproteomics elucidates active signaling dependencies and adaptive reconfigurations that should inform rational combination therapies and resistance-aware treatment strategies in CCA [14,18,19,53].

### 5.7. Cross-Platform Analysis Under the Framework of CCA

Sequencing technologies, when evaluated comparatively, exhibit superior sensitivity in identifying genetic alterations and reconstructing clonal evolution; however, they are constrained in their capacity to forecast functional signaling dependencies that influence treatment responses in CCA [57,64]. MS-based proteogenomics addresses this limitation by elucidating protein-level outcomes of genomic alterations and facilitating subtype-specific classification and biomarker identification; however, challenges such as standardization, tissue prerequisites, and analytical intricacy hinder its routine clinical application [14,15,18,19]. Phosphoproteomics introduces an essential functional dimension by elucidating activated kinase networks and adaptive resistance mechanisms; however, it is technically challenging and highly susceptible to pre-analytical variability. This underscores the necessity for integrative study designs that synchronize sampling strategies with the biological enquiries of intratumoral heterogeneity and therapeutic decision-making in cholangiocarcinoma [53,87,103].

## 6. Impact of Tumor Heterogeneity on Drug Resistance

ITH significantly contributes to medication resistance in cancer, particularly in CCA. The existence of many subclonal populations with unique genetic, epigenetic, and phenotypic traits within a single tumor complicates therapeutic interventions and frequently results in treatment failure. Both intrinsic and acquired drug resistance pose a substantial problem in cancer therapy, making it essential to comprehend the influence of tumor heterogeneity on this phenomenon to formulate successful treatment methods [5,104].

### 6.1. Mechanisms of Drug Resistance in Heterogeneous Tumors

Drug resistance in CCA is a multifaceted phenomenon resulting from both intrinsic and extrinsic tumor mechanisms, which can be classified into chemoresistance and resistance to targeted therapies, rather than being exclusively ascribed to evolutionary concepts like clonal evolution or phenotypic plasticity. Recent comprehensive assessments of biliary tract cancer indicate that resistance mechanisms are more precisely categorized according to molecular and cellular processes that directly hinder treatment efficacy or facilitate tumor survival under therapeutic pressure [105].

Chemoresistance in CCA is mostly caused by modified drug transport and metabolism, improved DNA damage repair, avoidance of apoptosis, and the activation of pro-survival signaling pathways. Dysregulation of ATP-binding cassette (ABC) transporters results in diminished intracellular drug accumulation, while enhanced detoxification capability and modified drug-metabolizing enzymes further restrict cytotoxic potency. Simultaneously, the activation of DNA repair pathways and deficiencies in apoptotic signaling enable tumor cells to endure chemotherapy-induced stress. These processes are often augmented by signaling pathways like PI3K/AKT, MAPK, and NF-κB, which enhance cell survival and facilitate inherent or acquired chemoresistance in biliary tract malignancies [105].

Resistance to targeted therapy in CCA adheres to specific yet interconnected principles, frequently mediated by both on-target and off-target mechanisms. On-target resistance involves secondary mutations or structural modifications in the drug target that diminish inhibitor binding, while off-target resistance results from the activation of compensatory or bypass signaling pathways that re-establish downstream signaling despite successful target suppression. Inhibition of receptor tyrosine kinases, such as EGFR or FGFR, may result in compensatory activation of other pathways, including MET, HER2, or downstream MAPK signaling, thus maintaining proliferative and survival signals. Furthermore, route redundancy and feedback reactivation are prevalent characteristics that restrict the efficacy of targeted therapeutics in CCA [105].

The tumor microenvironment significantly influences both chemoresistance and resistance to targeted therapy. Cancer-associated fibroblasts, immune cells, hypoxic environments, and inflammatory cytokines establish a protective niche that fosters survival signaling, metabolic adaptability, and treatment resistance. Extrinsic variables interact with tumor-intrinsic resistance mechanisms, leading to spatial and temporal variation in treatment responses across tumor locations [105].

In this perspective, intratumoral heterogeneity should be regarded as a result and enhancer of these molecular resistance mechanisms rather than a primary mechanistic category. Integrative proteogenomic and phosphoproteomic analyses are crucial for elucidating the activation of various resistance pathways in tumor subclones and microenvironmental niches, thereby establishing a functional framework for identifying rational combination therapies and addressing drug resistance in CCA.

Drug resistance in heterogeneous tumors is propelled by various mechanisms, including clonal evolution, phenotypic plasticity, and the adaptive responses of cancer cells to therapeutic selective pressures. In the clonal evolution paradigm, pre-existing subclones possessing mutations that confer resistance to a particular medication are selectively amplified during treatment. These resistant subclones may subsequently proliferate, resulting in tumor recurrence and advancement. Mutations in the TP53 and KRAS genes, commonly found in CCA, are linked to resistance against chemotherapy and targeted treatments [106,107].

Phenotypic plasticity, the capacity of cancer cells to transition among several functional states, is also essential in treatment resistance. In CCA, epithelial-to-mesenchymal transition (EMT) is a recognized process that enhances cellular plasticity. During epithelial–mesenchymal transition (EMT), epithelial cancer cells develop mesenchymal characteristics, such as enhanced motility, invasiveness, and resistance to apoptosis. This transition frequently involves modifications in the expression of critical signaling proteins, potentially affecting the sensitivity of cancer cells to targeted therapy. The dynamic characteristics of epithelial–mesenchymal transition enable cancer cells to swiftly adapt to treatment stresses, resulting in the development of drug-resistant phenotypes [108,109,110].

### 6.2. Role of the Tumor Microenvironment in Drug Resistance

The tumor microenvironment (TME) profoundly affects the emergence of medication resistance in CCA. The tumor microenvironment comprises diverse stromal cells, immune cells, and extracellular matrix elements that engage with cancer cells, forming a protective niche that facilitates tumor survival and adaptation. Cancer-associated fibroblasts (CAFs), integral to the tumor microenvironment (TME), release growth factors and cytokines that activate pro-survival signaling pathways in cancer cells, therefore fostering resistance to targeted therapies. In CCA, the activation of the PI3K/AKT and MAPK pathways by proteins produced by CAFs has been associated with diminished susceptibility to inhibitors directed at these pathways [38,111,112].

Hypoxia, a prevalent characteristic of the tumor microenvironment, exacerbates drug resistance by prompting metabolic adaptations and modifying gene expression profiles in cancer cells. Hypoxic environments stabilize hypoxia-inducible factors (HIFs), which govern the transcription of genes associated with angiogenesis, glucose metabolism, and cellular survival. The activation of HIFs has been linked to enhanced resistance to chemotherapy and radiation in CCA. Hypoxia-driven signaling promotes a more aggressive and resilient phenotype, hence enhancing the survival of cancer cell subpopulations capable of withstanding treatment stresses [98,113,114].

### 6.3. Adaptive Resistance and Compensatory Signaling Pathways

Adaptive resistance, defined by the activation of compensatory signaling pathways in reaction to treatment, is a significant factor contributing to drug resistance in heterogeneous cancers. In CCA, the blockage of a particular oncogenic pathway frequently results in the activation of other pathways that circumvent the therapeutic barrier. Targeting the EGFR pathway may lead to the activation of the MET or HER2 pathways, offering an escape mechanism for cancer cells and diminishing the efficacy of EGFR inhibitors [107,115].

Phosphoproteomic investigations have underscored the intricacy of adaptive resistance mechanisms in CCA, demonstrating dynamic alterations in phosphorylation patterns subsequent to targeted therapy. These modifications indicate the activation of compensatory mechanisms that allow cancer cells to persist despite the initial suppression of critical signaling pathways. The detection of adaptive alterations via phosphoproteomics can inform the development of combination medicines that concurrently target numerous pathways, potentially surmounting resistance and enhancing treatment effects [109,116].

### 6.4. Implications for Combination Therapy

Considering the influence of intratumoral heterogeneity on drug resistance, combination therapy has surfaced as a viable technique to tackle this issue. Combination therapy can diminish the probability of resistance and efficiently eradicate various subclonal populations within the tumor by concurrently targeting numerous signaling pathways. In CCA, the combination of PI3K/AKT pathway inhibitors with MAPK pathway-targeting drugs has demonstrated synergistic effects, enhancing the total response and postponing the development of resistant clones [51,114,117].

The efficacy of combination therapy depends on a comprehensive understanding of the tumor’s inherent heterogeneity. Proteogenomic methodologies, which amalgamate proteomic data from mass spectrometry with genomic and transcriptome insights, provide a robust framework for pinpointing the principal determinants of resistance and finding appropriate targets for combination therapy. This integrative concept facilitates a personalized approach to cancer treatment, customized to the distinct biological characteristics of each patient’s tumor [18,32].

### 6.5. Future Directions

Combating medication resistance in CCA necessitates a holistic strategy that accounts for the tumor’s dynamic and varied characteristics. Progress in single-cell sequencing, multi-region sampling, and liquid biopsy is anticipated to yield more comprehensive understanding of tumor clonal architecture and the development of drug-resistant subclones. Furthermore, the integration of phosphoproteomics with other omics data will augment our comprehension of adaptive resistance mechanisms and guide the formulation of innovative therapeutic methods capable of efficiently targeting the varied cellular populations inside CCA [34,64,75,118].

Utilizing these advanced technologies and implementing a multi-targeted strategy may enable the mitigation of challenges presented by tumor heterogeneity, hence enhancing clinical outcomes for CCA patients.

## 7. Proteogenomics Applications in Cholangiocarcinoma

Proteogenomics, the comprehensive study of proteomic and genomic data, has emerged as a potent method for exploring the intricate molecular landscape of malignancies, including CCA. Proteogenomics integrates mass spectrometry-based proteomics with NGS to offer an extensive analysis of the tumor’s molecular profile, correlating genomic mutations with their functional implications at the protein level. This integrative approach has demonstrated efficacy in revealing novel biomarkers, comprehending tumor heterogeneity, and pinpointing prospective treatment targets in CCA [24,119].

### 7.1. Identifying Novel Protein Variants and Biomarkers

A key application of proteogenomics in CCA is the detection of new protein variations resulting from somatic mutations, alternative splicing events, and gene fusions. These protein variations, frequently imperceptible via conventional genomic analysis, can function as significant indicators for diagnosis, prognosis, and therapy response. Proteogenomic investigations in CCA have identified novel isoforms of critical signaling proteins, including FGFR2 and EGFR, which are often modified in CCA and are integral to tumor growth and drug resistance [120,121].

Proteogenomics has enabled the discovery of novel protein variations and the identification of differentially expressed proteins and post-translational modifications (PTMs) linked to specific genetic changes. Tumors with IDH1 mutations display unique proteome profiles marked by modified expression of metabolic enzymes and phosphorylation of critical signaling proteins. These findings elucidate the metabolic reprogramming linked to IDH1 mutations and indicate potential metabolic vulnerabilities for therapeutic targeting [122,123].

### 7.2. Uncovering Mechanisms of Tumor Heterogeneity

ITH is a defining characteristic of CCA and a significant contributor to treatment resistance. Proteogenomics has been essential in determining the molecular foundation of this variability, uncovering the intricate relationship between genetic modifications and protein expression. Through the integration of multi-omics data, researchers have delineated multiple molecular subtypes of CCA, each characterized by distinctive proteomic and genomic fingerprints, which correlate with varying clinical outcomes and therapeutic responses [124,125].

Multi-region proteogenomic investigations have elucidated the spatial heterogeneity of CCA. Research indicates that various locations within a single tumor might display heterogeneous proteomic profiles, signifying the existence of many subclonal populations with unique functional attributes. This geographical heterogeneity significantly impacts therapeutic decision-making, indicating that a single biopsy may not fully represent the genetic diversity within the tumor [126,127].

### 7.3. Mapping Dysregulated Signaling Pathways

Proteogenomics has demonstrated significant efficacy in delineating the dysregulated signaling pathways that propel CCA development. By correlating proteomic data with genomic changes, researchers might discern critical oncogenic pathways activated in particular tumor subtypes. Proteogenomic study has elucidated the significance of the PI3K/AKT/mTOR and MAPK pathways in CCA, with their hyperactivation being correlated with unfavorable prognosis and resistance to targeted therapy [92,128].

Phosphoproteomics, a branch of proteogenomics dedicated to the examination of phosphorylated proteins, has proven particularly beneficial in elucidating active signaling networks in CCA. Phosphoproteomic studies have identified novel regulatory mechanisms and feedback loops contributing to treatment resistance by mapping phosphorylation sites throughout the proteome. The identification of compensatory phosphorylation events in response to PI3K inhibitors has guided the development of combination medicines targeting numerous pathways concurrently [129].

### 7.4. Guiding Personalized Therapy

The use of proteogenomic data could transform the creation of individualized treatment regimens in CCA. Proteogenomics offers a comprehensive molecular profile of each patient’s tumor, facilitating the identification of actionable mutations and protein biomarkers that inform the choice of targeted therapy. This method has been employed in clinical environments to categorize patients according to the molecular attributes of their tumors, facilitating more accurate and effective treatment strategies [83].

In patients with FGFR2 fusions, proteogenomic research has shown unique protein expression patterns linked to the fusion event, including the overexpression of downstream signaling pathways including ERK and STAT3. These findings have guided the application of FGFR inhibitors in clinical studies, showing substantial therapeutic advantages for patients with FGFR2-altered CCA. The identification of resistance mechanisms via proteogenomic profiling has facilitated the creation of next-generation inhibitors aimed at overcoming resistance and enhancing patient outcomes [94,130].

### 7.5. Future Directions in Proteogenomics for CCA

Proteogenomics is progressing swiftly, with continuous advancements in mass spectrometry equipment, data gathering techniques, and computational analysis tools. Innovative methodologies like single-cell proteogenomics and spatially resolved proteomics offer enhanced resolution in examining tumor heterogeneity, elucidating the molecular intricacies of CCA to an unparalleled extent [131,132].

Future studies will likely concentrate on the integration of proteogenomic data with additional omics layers, like as metabolomics and transcriptomics, to develop a more holistic picture of the tumor ecosystem. This comprehensive approach will improve our comprehension of the relationships among various molecular networks and create new options for identifying therapeutic targets. Utilizing proteogenomics enables a more profound understanding of CCA biology, hence facilitating the advancement of more effective, individualized cancer treatments [18,133].

### 7.6. Future Directions and Clinical Implications

The swiftly evolving domain of proteogenomics presents considerable potential for enhancing our comprehension of CCA and boosting patient outcomes via tailored treatment. Proteogenomics integrates proteomic, genomic, and transcriptome data to offer a comprehensive perspective on tumor biology, elucidating the intricate interactions between genetic changes and their functional implications at the protein level. This methodology has produced significant insights into the molecular determinants of CCA; nevertheless, other critical domains still require investigation as the discipline progresses [134,135].

### 7.7. Integration of Multi-Omics Approaches

Future investigations in CCA are anticipated to concentrate on the amalgamation of multi-omics data, including metabolomics, epigenomics, and single-cell analysis, to construct a thorough molecular map of the tumor ecosystem. Proteogenomics has established a robust framework for correlating genomic modifications with variations in protein expression; nevertheless, the integration of supplementary omics layers will yield a more comprehensive understanding of the molecular networks that underpin tumor heterogeneity. Integrating metabolomics can clarify the metabolic reprogramming linked to specific genetic abnormalities, such as IDH1, offering new insights into possible treatment targets [136,137].

Single-cell proteogenomics is a novel methodology designed to elucidate cellular heterogeneity within tumors with greater precision than bulk analysis. Through the analysis of individual cells, researchers can discern unusual subclonal populations that may play a role in medication resistance or tumor growth. This high-resolution research can uncover essential insights into the clonal architecture of CCA and inform the creation of tailored medicines designed to eradicate these aggressive subpopulations [138,139].

### 7.8. Advances in Mass Spectrometry Technology

Advancements in mass spectrometry (MS) are continually enhancing the potential of proteogenomics. Novel mass spectrometry technologies, characterized by enhanced sensitivity, superior resolution, and accelerated acquisition rates, provide more comprehensive proteome analysis and precise quantification of low-abundance proteins and post-translational modifications (PTMs). Furthermore, the advancement of data-independent acquisition (DIA) techniques and improved ion mobility spectrometry is anticipated to augment the depth and repeatability of proteome investigations [13,48,140,141].

The integration of spatially resolved proteomics represents a promising direction for future research. This method facilitates the examination of protein expression within the geographical framework of the TME, yielding insights into the interactions between cancer cells and adjacent stromal or immune cells. Comprehending these spatial correlations is essential for elucidating the mechanisms that govern intratumoral heterogeneity and affect treatment responses [12,142].

### 7.9. Clinical Implications of Proteogenomics

Proteogenomics may revolutionize CCA management by facilitating more accurate and individualized therapy approaches. Proteogenomics’ most promising element is its capacity to uncover actionable protein biomarkers associated with specific genetic abnormalities, thereby enabling the selection of targeted therapy customized to the molecular profile of each patient’s tumor [7,24].

In CCA, proteogenomic profiling has demonstrated potential in directing the application of FGFR inhibitors for patients with FGFR2 fusions. Proteogenomics has facilitated the stratification of individuals likely to benefit from targeted therapy by identifying specific protein expression patterns linked to FGFR2 changes, hence enhancing therapeutic results. This method also offers possibilities for monitoring therapeutic responses and identifying early symptoms of resistance, facilitating prompt modifications to treatment strategies [94,95].

Moreover, the elucidation of adaptive resistance mechanisms using proteogenomic investigations can guide the development of combination medicines that concurrently target several signaling pathways. The compensatory activation of the MAPK pathway in response to PI3K/AKT inhibitors has been noted in CCA, indicating that simultaneous inhibition of both pathways may surmount resistance and improve therapeutic success. By comprehending the dynamic alterations in signaling networks, proteogenomics can offer a framework for formulating sensible, multi-targeted therapeutic approaches [58,92,143].

### 7.10. Challenges and Opportunities

Notwithstanding the potential of proteogenomics, some hurdles persist that must be resolved to completely achieve its therapeutic efficacy. A significant challenge is the intricacy of data integration and analysis due to the vast volume of information produced by multi-omics investigations. Advanced computational tools and machine learning algorithms are essential for precise data interpretation, pattern identification, and actionable insight derivation. Cooperative endeavors among bioinformaticians, molecular biologists, and clinicians will be crucial for addressing these problems and using proteogenomic discoveries in clinical practice [16,135].

A further problem pertains to the standardization of proteogenomic procedures and the validation of biomarkers for therapeutic use. The repeatability of proteomic data may be influenced by discrepancies in sample preparation, data collecting, and analytical techniques. Establishing standardized methods and stringent quality control techniques will be essential for guaranteeing the reliability of proteogenomic tests in clinical environments [15,63].

### 7.11. Future Prospects in Personalized Medicine

The incorporation of proteogenomics into standard clinical practice could revolutionize the therapy paradigm for CCA and other malignancies. Proteogenomics offers a detailed molecular profile of each patient’s tumor, aiding in the discovery of new drug targets, allowing for patient stratification in clinical trials, and assisting in the creation of personalized treatment plans tailored to the unique molecular characteristics of each tumor. The utilization of liquid biopsies, in conjunction with proteogenomic analysis, provides a non-invasive method for the real-time observation of tumor dynamics, facilitating the early identification of resistance and the modification of therapy [64,144].

In summary, the future of cancer research and treatment depends on the ongoing advancement of integrative multi-omics methodologies, including proteogenomics. By using these new technologies, we can attain profound insights into the molecular foundations of CCA, hence facilitating the development of more effective, customized therapy options that enhance patient outcomes and quality of life.

## 8. Conclusions

CCA is a complicated and aggressive cancer characterized by considerable ITH, presenting significant hurdles for detection and treatment. Diverse subclonal populations, influenced by genetic mutations, epigenetic changes, and tumor microenvironment interactions, lead to treatment resistance and unfavorable clinical outcomes. Comprehending the factors that contribute to this variability is essential for formulating more effective therapeutic solutions [41,145].

Proteogenomics, an integrative methodology that merges mass spectrometry-based proteomics with NGS, has arisen as a potent instrument for clarifying the molecular landscape of CCA. Proteogenomics offers an extensive perspective on tumor biology by correlating genomic mutations with their functional protein products, facilitating the discovery of novel protein variations, biomarkers, and disrupted signaling pathways. This methodology has markedly improved our comprehension of the molecular determinants of CCA and has enabled the identification of actionable targets for precision therapy [146,147].

Proteogenomics applications in CCA research have shown significant potential, especially in informing individualized treatment approaches. Proteogenomic profiling has facilitated patient categorization according to distinct molecular traits, guiding the choice of targeted medicines and enhancing clinical outcomes. The capacity to identify adaptive resistance mechanisms has facilitated the development of rational combination medicines intended to surmount treatment resistance and improve efficacy [109,116].

The future combination of proteogenomics with additional multi-omics data, including metabolomics and single-cell sequencing, is anticipated to yield profound insights into the molecular foundations of CCA. Improvements in mass spectrometry technology and computer analysis tools will augment the depth and precision of proteome profiling, facilitating a more comprehensive evaluation of tumor heterogeneity. By adopting these creative methodologies, future cancer research can progress toward attaining a holistic, multi-faceted comprehension of tumor biology [148,149].

The clinical implications of proteogenomics are considerable, presenting new prospects for the creation of customized therapy methods that address the individual molecular aspects of each patient’s tumor. With the increasing integration of proteogenomics into clinical workflows, it has the capacity to transform cancer diagnoses, enable the discovery of new drug targets, and direct the use of more accurate and successful therapy strategies. The ongoing progression of proteogenomics in CCA research and its application in clinical practice offer significant potential for enhancing patient outcomes and furthering personalized oncology [18,24,150].

## Data Availability

No new data were created or analyzed in this study.

## Abbreviations

The following abbreviations are used in this manuscript:

AKT	AKT serine/threonine kinase
CAF	Cancer-associated fibroblast
CCA	Cholangiocarcinoma
ctDNA	Circulating tumor DNA
DIA	Data-independent acquisition
EGFR	Epidermal growth factor receptor
EMT	Epithelial-to-mesenchymal transition
FGFR	Fibroblast growth factor receptor
HIF1α	Hypoxia-inducible factor 1-alpha
IDH1	Isocitrate dehydrogenase 1
ITH	Intratumoral heterogeneity
KRAS	Kirsten rat sarcoma viral oncogene
MAPK	Mitogen-activated protein kinase
mTOR	Mechanistic target of rapamycin
NGS	Next-generation sequencing
PI3K	Phosphoinositide 3-kinase
PTM	Post-translational modification
RNA-seq	RNA sequencing
STAT3	Signal transducer and activator of transcription 3
TIL	Tumor-infiltrating lymphocyte
TME	Tumor microenvironment
TP53	Tumor protein p53
WES	Whole-exome sequencing
WGS	Whole-genome sequencing
